# Implementing a Non-Specialist Delivered Psychological Intervention for Young Adolescents in a Protracted Refugee Setting: a Qualitative Process Evaluation in Lebanon

**DOI:** 10.1007/s11414-023-09870-3

**Published:** 2023-12-12

**Authors:** Rayane Ali, Felicity L. Brown, Kerrie Stevenson, Mark Jordans, Karine Taha, Mounif El Amine, Frederik Steen, Bassel Meksassi, Joseph Elias, May Aoun, Bayard Roberts, Marit Sijbrandij, Pim Cuijpers, Aemal Akhtar, Aiysha Malik, Aniek Woodward, Daniela C. Fuhr, on behalf of the STRENGTHS Consortium

**Affiliations:** 1 Research and Development Department, War Child, Amsterdam, The Netherlands; 2 War Child Lebanon, Beirut, Lebanon; 3 Amsterdam Institute of Social Science Research, University of Amsterdam, Amsterdam, The Netherlands; 4 Department of Health Services Research and Policy, London School of Hygiene and Tropical Medicine, London, UK; 5 Institute of Health Informatics, University College London, London, UK; 6 Clinical, Neuro and Developmental Psychology, VU University, Amsterdam, The Netherlands; 7 School of Psychology, University of New South Wales, Sydney, NSW Australia; 8 Department of Clinical Neuroscience, Division of Insurance Medicine, Karolinska Institutet, Stockholm, Sweden; 9 Department of Mental Health and Substance Abuse, World Health Organization, Geneva, Switzerland; 10 KIT Royal Tropical Institute, KIT Health, Mauritskade 64, 1092 AD Amsterdam, The Netherlands; 11 Athena Institute, Amsterdam Public Health Research Institute, Vrije Universiteit Amsterdam, Amsterdam, Netherlands; 12 Leibniz Institute of Prevention Research and Epidemiology, Bremen, Germany; 13 Health Sciences, University of Bremen, Bremen, Germany

## Abstract

There has been an increase in the evaluation and implementation of non-specialist delivered psychological interventions to address unmet mental health needs in humanitarian emergencies. While randomized controlled trials (RCTs) provide important evidence about intervention impact, complementary qualitative process evaluations are essential to understand key implementation processes and inform future scaling up of the intervention. This study was conducted as part of an RCT of the Early Adolescents Skills for Emotions (EASE) psychological intervention for young adolescents with elevated psychological distress (predominantly with a Syrian refugee background) in Lebanon. Our aims were firstly to conduct a qualitative process evaluation to understand stakeholder experiences and perceived impact of the intervention and identify barriers and facilitators for implementation, and secondly to explore considerations for scaling up. Eleven key informant interviews and seven focus groups were conducted with 39 respondents including adolescent and caregiver participants, trainers, providers, outreach workers, and local stakeholders. Data were analyzed using inductive and deductive thematic analysis. Respondents perceived the intervention to be highly needed and reported improvements in adolescent mental health and wellbeing. Key implementation factors that have potential to influence engagement, adherence, and perceived impact included the socio-economic situation of families, mental health stigma, coordination within and between sectors (particularly for scaling up), embedding the intervention within existing service pathways, having clear quality and accountability processes including training and supervision for non-specialists, and sustainable funding. Our findings provide important context for understanding effectiveness outcomes of the RCT and highlights factors that need to be considered when implementing a mental health intervention on a larger scale in a complex crisis.

## Introduction

In 2022, there were more than 100 million forcibly displaced people worldwide, and almost half of these were children or adolescents.^1^ These young people are vulnerable to developing mental health problems due to exposure to armed conflict, migration, acculturation difficulties, poverty, and parental stress.^2–4^ It is estimated that one in five individuals in conflict-affected settings experiences a mental health disorder, compared to a global average estimate of one in 14, and the burden of these disorders is significantly higher than global averages across all age groups including children and adolescents.^5^ Compounding this risk, most (83%) refugees are hosted in low- and middle-income countries (LMICs) where there are significant challenges in providing adequate mental health and psychosocial support to address these mental health concerns (MHPSS)0.1,^6, 7^ Key barriers to service provision in LMICs include limited financial resources, under-resourced and unavailable mental health workforces, and stigma associated with mental illness.^6, 8, 9^

Due to these challenges, there is a recent push for the development and implementation of brief psychological interventions that can be delivered by trained and supervised non-specialists (those without specialist mental health training, such as community health workers) to increase the available mental health workforce, and thereby improve accessibility of evidence-based care. This “task-sharing” approach has demonstrated safety and effectiveness for addressing a range of mental health concerns in adults in LMICs,^10^ provided that adequate training, supervision, and support are ensured. For example, the World Health Organization (WHO) has developed several interventions to address psychological distress in adult populations living in settings of adversity.^11, 12^ More recently, WHO developed the Early Adolescent Skills for Emotions (EASE) intervention, a transdiagnostic intervention for young adolescents which aims to mitigate the symptoms of internalizing disorders such as depression and anxiety.^13^ It is a group-based intervention comprising seven 90-min sessions teaching young people coping skills to enhance psychological wellbeing, with three parallel sessions for caregivers. EASE has four core features: (i) brief in duration; (ii) delivered by non-specialist providers; (iii) transdiagnostic, addressing symptoms of depression, anxiety, and general distress; and (iv) designed for individuals affected by adversity (such as exposure to armed conflict).

This study is part of the STRENGTHS project, which aimed to evaluate community based mental healthcare interventions delivered by non-specialists to address psychological distress of Syrian refugees in eight countries across Europe and the Middle East.^7^ One of the sites was Lebanon, which hosts the largest number of refugees per capita in the world, including approximately 1.5 million Syrian refugees.^1^ Of these, 90% live in extreme poverty, and 53% of children aged 6–17 years are out of school.^14^ In addition, the COVID-19 pandemic hit the country at a particularly difficult time of escalating economic crisis and political fragility, with widespread protests commencing in October 2019, and rapid devaluation of the Lebanese pound. This worsening of the socioeconomic situation exacerbated unemployment, poverty, and availability of services in the country for refugee and host communities.^14^ Lebanon is heavily reliant on a privatized healthcare system and an under-resourced mental health workforce with just 1.26 psychiatrists per 100,000 population. Moreover, just 3% of the mental health workforce work solely in government-administered health facilities.^15^ Although various non-governmental organizations (NGOs) provide basic mental healthcare and work collaboratively with the government, there are significant gaps in care, particularly for refugee communities.^16^

Within the STRENGTHS project, EASE was culturally and contextually adapted, and a randomized controlled trial (RCT) was conducted to evaluate EASE with 10–14-year-old adolescents in Lebanon.^17, 18^ The trial was ended early due to disruptions related to the COVID-19 pandemic and political unrest, with a sample of 198 adolescents.^18^ Results from this under-powered data set indicated no differences between treatment and control groups; those adolescents receiving the EASE intervention and those receiving enhanced treatment as usual (consisting of single-session home-based psychoeducation) both showed improvements on the primary outcome of adolescent-reported distress, and various secondary outcomes.^18^ Conversely, a concurrent evaluation of EASE in Jordan did find a significant treatment effect on internalizing symptoms.^19^

Alongside this RCT in Lebanon, we conducted a process evaluation via qualitative interviews with EASE participants and key stakeholders. Process evaluations are recommended for conducting evaluations of complex interventions, in order to promote understanding of the dynamic interactions between human, technical, and contextual elements of a trial that lead to, and impact upon, the outcomes.^20^ These evaluations may be particularly important in humanitarian settings, which are characterized by complexity such as socio-political instability, limited resources, variety of actors implementing diverse services, and a multitude of dynamic stressors impacting on participants.^21–23^ Understanding the experience of participants and providers, and challenges and facilitators of implementation, is essential both to understand the processes by which trial outcomes were achieved, and to inform future implementation and ‘scalability’, or the potential for scaling up.^24^ While their importance is generally recognized, process evaluations are not uniformly conducted in RCTs of mental health and psychosocial support (MHPSS) interventions in humanitarian settings, and there is a lack of clear consensus amongst researchers as to the perceived aim and optimal methods of such evaluations.^21^

The aims of this study were firstly to understand participant and stakeholder experiences and perceived impact of EASE and identify barriers and facilitators of implementation in this trial, and secondly to explore considerations for scaling up — including benefits and challenges of expanding and integrating such interventions into existing service provision. The analysis was completed prior to learning the findings of the effectiveness analysis and therefore was independent of knowledge regarding treatment effect.

## Methods

### Design

This study was a qualitative process evaluation, nested within a larger RCT, involving a total of seven focus group discussions (FGDs) and 11 key informant interviews (KIIs) conducted with EASE participants, implementation staff, and stakeholders between October 2019 and December 2020. Ethical approval was given by the ethical review boards of the World Health Organization (#ERC.0003000) and St Joseph’s University in Beirut (#USJ.201724).

### Setting

The RCT was conducted in North and Akkar governorates of Lebanon, in largely agricultural areas bordering Syria and hosting a high population of refugees along with vulnerable Lebanese population. These areas have the highest rates of food insecurity in Lebanon, with many families reliant on social or cash assistance to meet basic needs.^25^ Intervention sessions and assessments with RCT participants were all conducted in community centers, with transportation provided to bring adolescents and caregivers from their homes to the appointments. Other key stakeholder interviews were held with professionals across Lebanon, via videoconference due to COVID-19-related restrictions.

### Respondents

The RCT included young adolescents of Lebanese, Syrian, or Palestinian background aged 10–14 years, who were identified as experiencing emotional distress via screening with the Pediatric Symptom Checklist.^18^ Additional inclusion criteria included being unmarried and having a caregiver to consent for participation. We attempted to interview EASE participants through purposeful convenience sampling for age, gender, and location. After EASE group implementation, endline assessments were conducted, and a sample of participants were invited to take part in the interviews. However, due to early completion of the RCT and restrictions on group-based data collection due to COVID-19 pandemic, and the resultant smaller number of completed EASE groups than anticipated in our sampling plan, we were only able to interview a total of 14 EASE participants including adolescent participants, and caregivers and adolescents who dropped out. All the adolescents and caregivers participating in KIIs and FGDs were of Syrian nationality (see Table 1). All non-specialist EASE providers (*n* = 10), trainers/supervisors (*n* = 2), and outreach staff (*n* = 6) from the study were interviewed. Seven key stakeholders were selected through purposeful sampling to attain a sample of key MHPSS actors from different regions in Lebanon, with extensive experience in planning or implementing MHPSS services, including individuals from the National Mental Health Program within the Ministry of Public Health, UN agencies at national and regional levels, and key international organizations. They were identified through a brief mapping of relevant actors conducted by the local study team and implementing organisation, and directly invited to take part by the study team.

**Table 1:** Outline of respondent characteristics for qualitative process evaluation

Type of participant	Description	Nationality	Interview type and number	Sex	Governorate	Topics covered
Adolescent EASE participants	Adolescents aged 10–14 years experiencing psychological distress, who took part in EASE	Syrian	2 FGD	4 males 4 females	North, Akkar	- Acceptability and relevance of EASE - Feasibility of EASE - Utility, perceived benefits of EASE, and possible negative impacts - Barriers and facilitators for implementation
Caregiver dropout participants	Caregivers of adolescents taking part in EASE	Syrian	3 KII	2 males 1 female	North, Akkar	
Adolescent dropout participants	Participants who started EASE but did not complete all sessions	Syrian	3 KII	2 males 1 female	North, Akkar	
Stakeholders	Stakeholders with significant experience in MHPSS service delivery, policy, and coordination through Health and Child Protection sectors. Either from INGOs, government, or UN agencies	Lebanese	5 KIIs 1 FGD	7 females	North, Beirut, Akkar	- Engagement and retention - Barriers and facilitators for implementation - Scale up considerations
EASE Trainers	Staff from the implementing NGO with specialist mental health training, who delivered EASE training and supervised providers	Lebanese	1 FGD	1 male 1 female	Beirut	- Fidelity and supervision - Scale up considerations
EASE Providers	Non-specialist providers with experience delivering MHPSS but without formal mental health training. They received 10 days of classroom based training in EASE, participated in a supervised practice cycle, and received weekly supervision while implementing EASE	Lebanese/Syrian	2 FGD	9 females 1 males	North,Akkar	- Acceptability and relevance of EASE - Feasibility of EASE - Engagement and retention - Fidelity and supervision - Utility, perceived benefits of EASE, and possible negative impacts - Barriers and facilitators for implementation
Outreach workers	Staff from NGO with outreach experience and community connection in the North and Akkar regions	Lebanese	1 FGD	2 females 4 males	Tripoli and Akkar	- Acceptability and relevance of EASE - Feasibility of EASE - Utility, perceived benefits of EASE, and possible negative impacts - Engagement and retention - Barriers and facilitators for implementation - Scale up considerations
Total			18 interviews 39 participants			

### Procedure

Written consent and assent to take part in the qualitative interviews was collected for EASE caregiver and adolescent participants respectively at their enrolment in the study. Verbal assent and consent was taken again prior to the interviews. For other respondents, written informed consent was collected before the interview. The interviews were conducted in Arabic for adolescents and caregivers, providers, and outreach workers and were offered in English or Arabic for stakeholders, upon the preference of each respondent.

FGDs and KIIs were conducted by one or two interviewers, supported by a note taker, and followed a semi-structured interview guide. The guides were developed collaboratively by research partners, informed by research on scale up of MHPSS interventions,^22, 24^ and translated into Arabic. Training was given to local research assistants who conducted the interviews, including qualitative research basics, child safeguarding and adverse events, using the guides, using the recorders, and taking field notes. With respondent consent, interviews were audio-recorded; however, one respondent declined and notes were taken by a note taker. Interviews were 60–90 min in duration.

### Data Analysis

The six English interviews were transcribed verbatim, while the 13 Arabic interviews were transcribed and translated from Arabic audio directly to English. This work was conducted by trained bilingual research team members (RA, BM, JE) and was quality checked by another team member for accuracy and completeness.

Prior to data analysis, a reflection workshop was conducted with the transcription team to learn about recurrent patterns and emerging themes they noted in the data. Data analysis was conducted via inductive and deductive thematic analysis.^26^ Two researchers (RA and FB) read the finalized transcripts to familiarize themselves with the data and separately open coded to identify and label any segments of data perceived to be relevant to the research questions. Two workshops were conducted to develop the codebook. In the first workshop, RA and FB compared their individual open coding and compiled one draft codebook where major categories of data were assigned labels (codes) and working definitions. In the second workshop, the Lebanon study team reviewed and revised the proposed codebook structure and content. A series of revisions were then conducted by a core analysis team (RA, KS, FB, DF) to iteratively finalize the codebook. RA applied this codebook to four transcripts, using NVIVO 12 software (released March 2012). This coding sample was checked by KS and discrepancies discussed together, with minor changes made to the codebook, to better fit the data. At the end of this stage, both coders reached consensus on the application of the codebook*.* RA coded the remaining transcripts, and codes were populated with data from the transcripts. Once all transcripts were coded, RA, KS, FB, and DF met to review coding and structure data into themes and sub-themes. Four main themes and 15 subthemes were identified through merging related codes, and these were subsequently reviewed for consistency, applicability, and comprehensiveness of themes. Labels and definitions were agreed for each theme, and illustrative quotes identified. Themes were reviewed and validated with the full study team, including those closely involved with implementation. During analysis of final transcripts, no major new ideas from transcripts emerged, suggesting that data saturation was reached.

## Results

**Figure 1.**
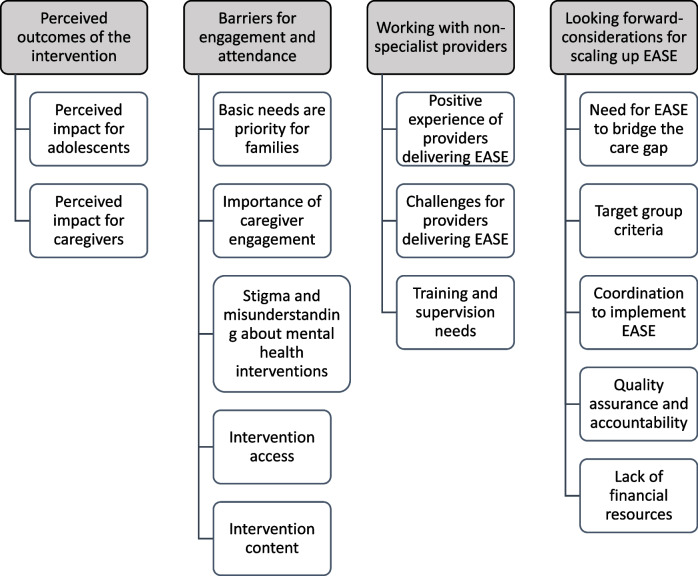
Tree of the four main themes and the 15 subthemes from the qualitative analysis

Four major themes and 15 subthemes were identified related to the implementation and potential for scale up of EASE in this setting. They are illustrated in Fig. 1 and outlined below, with illustrative quotes.

### Theme 1: Perceived Outcomes of the Program for Adolescents and Caregivers

Most respondents expressed their positive experiences of participating in EASE, either as intervention participants, providers, or trainers/supervisors. They appreciated the opportunity to attend an activity outside the home, and to share feelings and thoughts with others. One trainer described how “they were happy to have a space where they can go out and talk”. The majority of EASE participants, providers, and trainers/supervisors also reported significant benefits obtained for adolescents, caregivers, and relationships inside and outside the family.

#### Perceived Outcomes for Adolescents

Most adolescents, caregivers, and providers highlighted positive impacts on adolescents at different levels. At the individual level, adolescents’ behavior improved, particularly regulating their reactions when feeling angry. For example, one adolescent noted that the strategy of slow breathing helped him to remain calm and manage strong emotions.

The slow breathing helped us so much, many times, when I’m angry or upset from someone, I do it and it makes me very relaxed, I do it, if I’m angry I do it, if I’m nervous or scared I do it (adolescent, male).

One female caregiver noted improvements in her adolescent boy:

I felt that he started to play- he even started to go outside the house and come back, he didn’t use to go out- he used to stay inside the house for a month or 20 days… and before I told you that he always I mean cries and do.. no, now he is better, thank god

Some respondents also noted improvement in adolescent’s ability to express themselves; how they can talk about their feelings, their personal strengths, their problems and how to deal with them, their daily life, and their relationships with their communities. One adolescent girl noted:

“before EASE we didn’t know much about solving our problems on our own, and now we know how to solve them”. One provider noted: “There were two girls, they didn’t talk at all, not any word, no reaction, no eye expression, and after the third session, they talked and expressed considerably." (provider, female). At the family level, some caregivers noted improved caregiver-child relationships. These respondents noted that this extended to improvements in relationships with others in the community, including interacting with other children, and reduced bullying or aggression with peers.

#### Perceived Outcomes for Caregivers

Most providers noted positive outcomes regarding parenting behaviors. Several caregivers and providers noted that physical punishment remains an ingrained disciplinary technique. However, EASE taught alternative strategies which many caregivers considered helpful. Additionally, the majority of caregivers noted that they learned the rationale for giving praise and how to effectively deliver it. This was considered helpful, despite not being a well-known concept previously. One caregiver noted: “I stopped the violence, I approached them with nice words, with coping words” (caregiver, female).

Many providers and caregivers also described a positive effect on caregivers’ own wellbeing and behavior. For example, some caregivers used EASE strategies to improve their relationships and communication with their partners. They also applied the strategies of self-care to improve their overall well-being, for example: “They knew that they should have space (take a break) during the day to entertain a little about myself [have some time for themselves], maybe drink a coffee with a neighbor, walk a little…” (provider, female).

### Theme 2: Barriers for Engagement, Attendance, and Impact

#### Basic Needs are a Priority for Families

Outreach workers, stakeholders, providers, and participants stated the predominant barrier for engagement of adolescents and caregivers with EASE was the predominance of unmet financial and basic needs such as food and housing, where efforts to achieve these needs have made participants “exhausted, not only tired”. With the deterioration of the economic situation in Lebanon, families prioritized direct income generating activities rather than MHPSS interventions (i.e. EASE) that do not provide financial support. Competing scheduling priorities were a major issue for attendance, including families moving for work opportunities, adolescents attending school or work, adolescents or caregivers needing to care for younger children or other family members, or UN agency appointments including for financial support. Since EASE is group-based, scheduling around individual participants’ competing priorities was particularly difficult. “It’s the biggest difficulty we face, that the child doesn’t come when it’s agriculture season…when it’s potato season then no children come” (provider, female). One male caregiver whose adolescent did not complete the program expressed that: “If someone found work, it would be more important than the programme”. In focus group discussions, both outreach workers and providers stressed that recruitment of new participants became even more challenging with wide-scale protests commencing October 2019 causing closures (e.g. of schools, offices) and roads blocks, and subsequent further deterioration of the economic situation. In both male and female adolescent focus groups, participants cited the “roadblocks” as a major barrier causing adolescents to be “afraid” and they “maybe don’t want to go out of house”, or “the place he lives in”. Other barriers by adolescents included medical concerns, work, and other competing priorities (“they might be going to somewhere more important than this”), or lack of need “they might not be in need for it”. Unfortunately, several interviewees noted that the most vulnerable adolescents were even less likely to access the intervention, for example: “…Tripoli and Akkar, where EASE was implemented, have the highest rate of children involved in the worst forms of child labor, long hours in agriculture or on the streets, with those long working hours, they will not have time to attend EASE sessions.” (MHPSS stakeholder).

#### Importance of Caregiver Engagement

Despite the program being offered to adolescents, the consent of both caregivers was considered by respondents as essential for ensuring child attendance. Some caregivers prevented attendance for different reasons including lack of financial support, needing their child to work inside or outside the home, or not wishing their daughter to attend a center at the same time as males. Some outreach workers noted that some Lebanese caregivers refused participation due to feeling that the intervention was for Syrian adolescents. Again, financial considerations played a large role in caregivers preventing their children from taking part, for example, one provider described: “The biggest difficulty is that there’s no financial allowance, the most difficult thing is that the parent tells the child go to work instead of going to play and have fun” (provider, female).

#### Stigma and Misunderstandings About Mental Health Interventions

Most MHPSS stakeholders stressed the fact that the stigma around accessing mental health interventions is prevalent in the community, and that it affects engagement with interventions like EASE. Different respondents mentioned that it would be very important to have a combination of outreach strategies to present the project in a way that it is not perceived as a “clinical” mental health intervention. This highlights the importance of carefully framing EASE in the wider context of healthcare or other community-based interventions and ensuring it is embedded in routine service provision to make it less stigmatizing.

Stigma is an issue nationally, it’s an issue for all interventions related to mental health and psychosocial support. But I think when we don’t say mental health it’s even easier, for when we say psychosocial support, and when we frame it in the sense that the intervention is not in mental health. (MHPSS stakeholder).

Additionally, challenges of uptake of an intervention known to be for those with heightened distress is “…linked to the stigma of like other people in the community knowing that their children are taking part in such an intervention.” (MHPSS stakeholder).

On the other hand, at times, there were misunderstandings about the nature of the EASE intervention, with some providers, trainers/supervisors, and outreach workers noting that some adolescents believed they were coming to “play,” similar to other psychosocial activities they had attended. Along with MHPSS stakeholders, they emphasized the importance of explaining the intervention clearly during outreach.

#### Intervention Access

Finding suitable locations for intervention delivery that were close to family homes for easy access was challenging. Families in this study lived in underserved agricultural areas, resulting in long commutes to intervention venues. Buses were provided for transport; however, all adolescents attending one group were picked up at various locations which led to lengthy trips for some adolescents. Some adolescents did not mind the long drives; however, providers noted that it could impact the sessions starting on time and the performance of participants. Additionally, outreach workers and caregivers observed that it required adolescents and caregivers to be out of the home for considerable periods of time.

I received multiple complaints, from the people participating with us, that the distance is too far away for them, also the roads of [agriculture area] are hard, especially in winter, and the bus needs a lot of time to make their way there (outreach worker, male).

Furthermore, finding suitable and comfortable venues with sufficient space for intervention delivery was difficult, and at times, not all activities could be implemented as planned.

It’s a school style, floors, classes, and seats, you enter and you don’t feel that the child is comfortable to enter such a place (provider, female)

#### Intervention Content

Although overall the feedback on the content and structure of the intervention was positive, some aspects were noted to make it challenging for some adolescents and caregivers. Some interviewees felt that the illustrations and story book used in EASE were more suited to younger adolescents than older adolescents. Additionally, one illustration of a character perceived to be male sweeping the floor was perceived to be unrealistic of the setting. Providers and trainers/supervisors stressed that some components of the intervention were complicated for adolescents to grasp, such as explaining the “vicious cycle of unhelpful behaviors and emotions” and the “changing my actions” strategy for changing behaviors in a stepped approach. It was felt that more sessions and more trainings for providers would be needed to cover these strategies adequately with adolescents. Providers additionally reported that the caregiver sessions were very dense with information, which was challenging to deliver and at times caused boredom in caregivers.

### Theme 3: Working with Non-Specialist Providers

#### Positive Experience of Providers Delivering EASE

The majority of stakeholders stressed that non-specialist providers are well placed to deliver EASE, and caregivers and children described their experience in working with the providers as “good” and “respectful”. Trainers/supervisors reported that providers were delivering the intervention with high fidelity and competency. This indicates feasibility of using non-specialist providers, if there is a clear package of competency building, with strong supervision and follow-up. Some stakeholders reported that a major benefit for non-specialist providers is that they are “*less costly*” compared to specialists which may increase the scalability of the intervention to reach more people. In the focus group with providers, they reported positive experiences delivering EASE, with personal benefits including applying some of the strategies in daily lives like “*slow breathing*”. Moreover, added professional benefits included improving knowledge, enhancing skills, and contributing to career development.

In EASE, it’s the first time I work in such a program. Like detailed to this extent and very specific, then this is for sure, this is so [..] positive for my professional experience (provider, female).

#### Challenges for Providers

Several role-related challenges were highlighted. Some providers felt that at times, they were unable to meet the needs and expectations of some participants, as they understood providers as trained mental health professionals. Additionally, due to the delivery model, providers were employed on a casual contract basis. Therefore, working hours depended on the schedule of intervention groups, which could be inconsistent and difficult to plan for, leading to frustrations amongst providers. Providers expressed the need for stability in employment and progression in careers, with other stakeholders also stressing that non-financial incentives such arenon-specialist providers. Stakeholders also reported the possibility for providers to be “*burned out*” (MHPSS Stakeholder,) in these roles, as they are working with distressed young adolescents, and emphasized the importance of promoting self-care amongst providers.

#### Training and Supervision Needs

The EASE training package was perceived by providers and trainers/supervisors to be notably different from other MHPSS trainings. The strong focus on role plays was perceived to soundly prepare providers for eventualities in sessions and enhance their problem-solving skills for challenging scenarios. The focus on individual provider competencies, in terms of highlighting existing strengths and focusing on further development of specific competencies when needed, was considered highly effective. Some respondents advised that additional trainings could be helpful on topics including types of distress adolescents experience, communication skills, and complaint and reporting mechanisms. Furthermore, respondents reported that rather than one intensive 10-day training, it may be more effective if training were spaced out to make it less tiring for providers and trainers.

Many providers, trainers/supervisors, and MHPSS stakeholders stressed that regular group supervision sessions were important and allowed providers to share their experiences with a specialist supervisor. In parallel, individual coaching or follow-up with providers is needed. Variation in strategies used during supervision (e.g., role-plays) was considered helpful.

Individual coaching or the individual follow up is needed. So the supervision sessions do not mean if I’m doing the [group] supervision sessions, that doesn’t mean I need to make the individual follow up less (MHPSS stakeholder)

### Theme 4: Looking Forward-Considerations for Scaling Up EASE

While the previous themes and sub-themes investigate implementation factors that can facilitate or hamper intervention delivery, this theme focusses specifically on findings regarding planning for future scale up of EASE in Lebanon, including structural and contextual challenges.

#### Need for EASE to Bridge the Care Gap

Interviewees, including providers, outreach workers, trainers/supervisors, and stakeholders, stressed that there is a significant need for focused psychosocial support services like EASE for children and adolescents in Lebanon.

EASE was reported to be well designed to meet this need, particularly due to the different levels of distress that people face in Lebanon. The recent successive crises (economic crisis, COVID-19 pandemic, and Beirut blast) have impacted basic needs, security, mental health, and wellbeing of the whole population. This has exacerbated the need for MHPSS interventions.

Now regarding what is happening with Lebanon, it is being crises, political and everything I mean, I feel EASE, it is the time now (outreach worker, female)

#### Target Group Criteria

When reflecting on who EASE should be offered to, there appeared to be a consensus that EASE should be delivered to young adolescents of all nationalities and not focused only on refugee or migrant communities, given the stressors that all young people in Lebanon are facing. Furthermore, some respondents emphasized the need to ensure the inclusion of the most vulnerable adolescents, including working adolescents, those living in Palestinian camps, and those with disabilities. Respondents noted that youth are most impacted by the country’s decline, and stakeholders stressed the need to expand the age range of EASE, considering the mental health needs of older adolescents and young adults, although this may require substantial adaptation of the EASE intervention.

#### Coordination to Implement EASE

The majority of interviewed stakeholders stressed that EASE “cannot be a stand-alone” intervention (MHPSS stakeholder, female). EASE has to be complementary to other provided services such as educational, recreational, or health care packages. Different interviewees stated that it is important to embed EASE delivery within “structures that are sustainably available” (MHPSS stakeholder, female), such as training at local universities, and delivering in schools, primary healthcare or community mental health centers, grass root organizations, and the referral pathways at the national level. EASE implementation should be in coordination with the overall mental health system in the country, child protection actors, Ministries of Public Health and Social Affairs, and the MHPSS Taskforces. Given the rapidly changing context in Lebanon, the need is not only to implement such an intervention, but also to maintain it longer term, and this would require coordination with other services. Additional support for adolescents requiring more specialized services may be needed as well, and this would require working together with tertiary providers. Some stakeholders additionally emphasized that integrating EASE with existing health or other services is a way to avoid stigma surrounding mental illness.

#### Quality Assurance and Accountability

Some respondents stressed the importance of having a system in place to ensure quality implementation and accountability and provided examples of minimum activities needed including regular training on relevant topics and competencies for providers; strong supervision with a specialist supervisor; regular coordination meetings and support for the implementation team; clear processes and guidelines, standard operating procedures, terms of references for each role; clear framework for responding when any responsibilities are breached; monitoring tools for the intervention; and clear reporting lines. Providers felt that having a “monitoring system with complaint mechanisms” is important, alongside setting key performance indicators to monitor and deliver feedback for them in their work.

[For] the intervention to sustainably be maintained with quality, there should be a system around it. There should be a system that guarantees proper training of these providers, proper support (MHPSS Stakeholder)

#### Lack of Financial Resources

Most stakeholders and trainers/supervisors emphasized that scaling up is likely to be hindered by lack of financial resources. Government funding for mental health is a major challenge in the context of the economic crisis and political situation, with many competing priorities. International mental health funding is limited, and not a sustainable solution. However, some stakeholders were optimistic and mentioned that the worsening situation in Lebanon might lead to more international interest and funding opportunities for MHPSS interventions including EASE. Nonetheless, lack of sustainable funding pathways was emphasized as a major barrier to scaling up EASE.

## Discussion

Overall, respondents reported that the EASE intervention was highly needed in Lebanon, and perceived that it led to improvements in adolescent mental health and wellbeing, specifically in relation to the ability to identify and regulate emotions and behavior, and strengthening interpersonal relationships. However, several intervention-specific and context-specific barriers to delivery were identified that were perceived to have negatively impacted engagement, attendance, and impact of EASE in this study, and have implications for its scalability. These included the following: (i) pervasiveness of unmet basic needs and the impact on adolescent and caregiver ability to engage in an MHPSS intervention; (ii) stigma regarding mental health impacting on engagement and retention; (iii) the challenges of ensuring adequate access to EASE, particularly for those living in agricultural areas, and; (iv) ensuring intervention materials are perceived relevant for all participants. Looking forward to what would be needed in future scale-up efforts, respondents emphasized (i) ensuring strong provider training and supervision; (ii) importance of coordination between and within sectors and integration into existing referral pathways to ensure adequate uptake and ability to meet additional needs of participants; (iii) ensuring a strong quality assurance and accountability system; and (iv) identifying sustainable funding sources. The need for EASE to be available and responsive to needs of vulnerable groups was stressed; future implementation work should consider applicability and effectiveness with these populations. Our findings emphasize the importance of considering broader macro-level influences on the potential reach and impact of the intervention.

Results of this study are important both in terms of contextualizing effectiveness results from the RCT within which this process evaluation was embedded, and for informing future research and implementation of EASE and other MHPSS interventions. The RCT was conducted under very difficult and unpredictable circumstances including a period of deteriorating economic and socio-political crises and associated growing social unrest in Lebanon, with 88% of Syrian households living under the extreme poverty line by 2021.^14^ Ultimately, it was ended early due to the COVID-19 pandemic and restrictions on implementing face to face interventions. However, exploratory quantitative results of the under-powered RCT indicated that adolescents receiving EASE did not improve significantly more than a control group. Seventy percent of adolescents attended five or more (out of seven) sessions, and 13% of adolescents did not attend any sessions.^18^

Our findings emphasize the major impact that the economic situation and frequent protests and roadblocks had on many families’ ability to engage with and benefit from EASE, and the increased likelihood that adolescents would be engaged in paid or unpaid labor to support the household, therefore being unable to attend EASE. Economic stress is a known social determinant of mental ill health^27^ and can impact on children via poor parental mental health and disrupted parenting.^28^ Our findings suggest that beyond increasing stress directly, poverty also impacts the ability to access and attend MHPSS interventions and increases risk through exposure to additional adversity such as child labor.

These circumstances are unfortunately not unique to Lebanon, with socio-political instability, conflict, and escalating poverty being ubiquitous in humanitarian emergencies. Global guidelines for conducting MHPSS in emergencies highlight the importance of multi-sectoral, integrated programming that ensures that holistic needs are met, including financial vulnerability and extensive protection risks^29^; however, this remains a challenge to successfully implement in extremely low resource settings in times of crisis. Future research is needed to identify the best ways to integrate MHPSS interventions with other multisectoral activities, such as financial poverty alleviation interventions to break negative cycles between poverty and poor mental health,^30^ and optimal timing of interventions.

Integrated interventions are also important to combat stigma. Respondents in this study emphasized the potential for integrated programming to overcome barriers to attendance that are related to mental health stigma, as interventions are then not perceived as solely for mental illness. Several others studies have indicated that stigma related to mental health and mental health treatment is common in Lebanon,^17^ and other humanitarian settings,^9^ and can deleteriously affect scale-up of psychological interventions in humanitarian and refugee resettlement contexts.^31, 32^ Therefore, determining the most effective pathways via which to best engage communities to avoid fears of stigma is essential, alongside developing culturally appropriate interventions for reducing stigma.^33^

Non-specialist provided psychological interventions are often conducted in group-based format, which is generally considered to be lower cost, increase availability, and provide social benefits. Our findings indicate that in this setting, there were several challenges associated with group-based delivery: (i) anonymous participation is not possible, (ii) suitable locations for delivery were difficult to identify and transportation was difficult, and; (iii) scheduling session times became more challenging. Implementation science studies, which include comparisons between group-based and individually delivered MHPSS interventions in humanitarian settings on cost effectiveness, attendance, and feasibility, will help advance understanding of optimal delivery formats.

Ultimately, a lay-delivered psychosocial intervention is built on a rigorous training and supervision scheme, involving theoretical and practical training and regular supervision. In order for this structure to be sustainable, studies in a variety of settings show that long-term funding and cooperation with local non-governmental organizations and the national government will be required.^34^ Qualitative studies considering scale-up of non-specialist provided interventions with adults in Jordan^32^ and the Netherlands^31^ have both highlighted the need for clear professional accreditation pathways for providers, meeting local legal requirements, as well as ensuring sustainable local funding mechanisms.

This process evaluation provides important insights on factors that need to be considered when implementing an MHPSS intervention in a complex crisis; however, our findings must be considered in the context of some limitations. First, we would have liked to interview additional adolescents and caregivers, but this was prohibited due to COVID-19 restrictions. However, we are confident that theoretical saturation was reached with the number of interviews that were conducted. Additionally, we had hoped to interview adolescent participants who had dropped-out of the EASE intervention to understand their experience. Unfortunately, we were only able to interview two of such participants, and their interviews provided limited information on challenges they experienced. Combined with a relatively small sample size overall, these challenges limit our confidence in the generalizability of findings to other interventions or settings.

## Implications for Behavioral Health

While the number of RCTs conducted in LMICs is increasing, to date, there remain few RCTs of behavioral health interventions in humanitarian settings, particularly with children.^35^ Given the contextual complexity that characterizes these settings, and the impact this can have on pathways between intervention delivery and impact, there is an urgent need to conduct further RCTs of child and adolescent interventions in humanitarian settings. Equal attention should be paid to the implementation process including factors enhancing or inhibiting impact and best practices for scale-up. This is especially important for the delivery of behavioral health interventions that are conducted in complex settings which are characterized by contextual influences that may be unforeseen. Our findings highlight the importance of attending to: (i) barriers for engagement, (ii) stigma relating to mental health interventions, (iii) integrating into holistic intersectoral approaches for refugee families to promote uptake and ensure other needs are met, (iv) ensuring strong quality implementation and accountability, and (v) sustainable funding mechanisms. Further research and engagement with practitioners and policy makers is crucial to design solutions for these system-level challenges in mental health service delivery in complex settings.

Use of implementation frameworks (such as RE-AIM^36^) can help intervention researchers to predict, understand, and document some of these factors to some extent, and would facilitate knowledge production and sharing amongst researchers and practitioners working in humanitarian settings. However, essential for all research, but even more so for process evaluations, co-producing research with local researchers and practitioners will ensure that intricate interactions between contextual, cultural, technical, and implementation factors are adequately captured and understood. Frameworks for scaling up are useful to plan for further maintenance and long-term sustainability after the project has ended. Ultimately, sustainable dissemination of the intervention requires close collaboration and coordination between different stakeholders ensuring that the financial, legal, and human resources are in place for successful long-term implementation.
